# Protein Inhibitor of Activated STAT3 Suppresses Oxidized LDL-induced Cell Responses during Atherosclerosis in Apolipoprotein E-deficient Mice

**DOI:** 10.1038/srep36790

**Published:** 2016-11-15

**Authors:** Rong Wang, Yanjin Zhang, Liran Xu, Yan Lin, Xiaofeng Yang, Liang Bai, Yulong Chen, Sihai Zhao, Jianglin Fan, Xianwu Cheng, Enqi Liu

**Affiliations:** 1 Research Institute of Atherosclerotic Disease, Xi’an Jiaotong University Cardiovascular Research Center, Xi’an, Shaanxi 710061, China; 2 Laboratory Animal Center, Xi’an Jiaotong University Health Science Center, Xi’an, Shaanxi 710061, China; 3 Molecular Virology Laboratory, VA-MD College of Veterinary Medicine and Maryland Pathogen Research Institute, University of Maryland, 8075 Greenmead Drive, College Park, MD 20742, USA; 4 Department of Molecular Pathology, Interdisciplinary Graduate School of Medicine, University of Yamanashi, Yamanashi 409-3898, Japan; 5 Department of Cardiology, Yanbian University Hospital, Yanji, Jilin 133000, China

## Abstract

Atherosclerosis is a serious public health concern. Excessive inflammatory responses of vascular cells are considered a pivotal pathogenesis mechanism underlying atherosclerosis development. It is known that Janus kinase/signal transducer and activator of transcription 3 (JAK/STAT3) signalling plays an important role in atherosclerosis progression. Protein inhibitor of activated STAT3 (PIAS3) is the key negative regulator of JAK/STAT3 signalling. However, its effect on atherogenesis is unknown. Here, we observed that PIAS3 levels are reduced in atherosclerotic lesions and that PIAS3 expression decreases in conjunction with increases in interleukin-6 expression and atherosclerosis severity. Oxidized low-density lipoprotein (ox-LDL), an atherogenic stimulus, reduced PIAS3 expression, an effect that may be attributed to nitric oxide synthesis upregulation. In turn, PIAS3 overexpression effectively suppressed ox-LDL-induced inflammation, lipid accumulation and vascular smooth muscle cell proliferation. These results indicate that PIAS3 is a critical repressor of atherosclerosis progression. The findings of this study have contributed to our understanding on the pathogenesis of atherosclerosis and have provided us with a potential target through which we can inhibit atherosclerosis-related cellular responses.

Atherosclerosis, a commonly pathological basis of cardiovascular disease, seriously threatens human health. Increasing amounts of evidence indicate that inflammatory responses play prominent roles in atherosclerosis initiation and development^1^,^2^,^3^. In aortic lesions, inflammatory cytokines, such as interleukin-6 (IL-6) and interferon γ, are significantly upregulated^4^,^5^. Both of these cytokines stimulate Janus kinase/signal transducers and activators of transcription (JAK/STAT) signalling pathway to promote vascular cell inflammation, proliferation, migration and adhesion^6^,^7^,^8^,^9^.

JAK/STAT signalling pathway is one of the stress response signalling pathways. Many stimuli that cause vascular stresses, such as angiotensin II, mechanical stress, oxidative stress, and IL-6/gp130 inflammation stimuli, induce JAK/STAT signalling activation^10^. IL-6 is a crucial inflammatory stimulus. Upon binding to cells, IL-6 forms a complex with IL-6 receptor α and gp130, thereby triggering JAK activation and STAT3 phosphorylation. Phosphorylated STAT3 then dimerizes and translocates into the nucleus to activate target gene expression, resulting in cell proliferation, cell survival, and immune responses^8^,^9^. Clinically, high-level IL-6 expression is generally considered an important marker of cardiovascular inflammation. During atherosclerosis, abundant IL-6 expression is detected in macrophages, T cells, endothelial cells, and vascular smooth muscle cells (VSMCs)^11^. Therefore, the IL-6/gp130-JAK-STAT3 signalling pathway plays a crucial role in inflammatory responses during atherogenesis.

Oxidized low-density lipoprotein (ox-LDL) is a well-known atherogenic factor and contributes to atherosclerotic plaque formation and progression by promoting inflammation, macrophage foam cell formation, and smooth muscle cell migration and proliferation^12^. Early studies demonstrated that ox-LDL is also able to activate STAT1 and STAT3, the transcription factors that mediate the effects of cytokines and growth factors^13^.

Excessive JAK/STAT signalling activation can lead to disordered immune responses and induce inflammatory damage. To avoid excessive inflammation *in vivo*, some protein inhibitors, such as suppressor of cytokine signalling (SOCS), protein inhibitor of activated STAT (PIAS) and protein tyrosine phosphatases, are involved in negatively regulating JAK/STAT signalling activation^14^. The findings of previous studies indicate that SOCS1 and SOCS3 are closely correlated with atherosclerosis progression^15^,^16^,^17^. High SOCS1 and SOCS3 expression levels were detected in VSMCs and macrophages in both human atherosclerotic plaques and apolipoprotein E knockout (ApoE^−/−^) mouse aortic lesions. SOCS proteins suppress STAT activation and reduce inflammatory gene expression and cell growth.

PIAS3 is the other pivotal negative regulator of JAK/STAT3 signalling. It can effectively inhibit IL-6/gp130-JAK-STAT3 signalling activation^18^. However, the involvement of PIAS3 in atherosclerosis development has not yet been defined. Thus, the objective of this study was to analyse PIAS3 expression and its roles in cellular responses during atherosclerosis. First, we assessed PIAS3 expression and its correlations with JAK/STAT3 signalling activation and inflammatory responses in the aortic lesions of ApoE^−/−^ mice. Then, we used the atherogenic stimuli IL-6 and ox-LDL to stimulate cultured cells to assess PIAS3 expression in the setting of atherosclerosis-induced inflammation. In addition, we investigated the effects of PIAS3 on atherosclerosis-related cellular responses, including inflammatory cytokine expression, foam cell formation and cell proliferation, and also examined the potential mechanisms underlying these processes. We found that PIAS3 is inversely correlated with atherosclerosis progression and is a repressor for atherosclerosis-related cellular responses. These findings suggest that PIAS3 might be a potential target through which atherosclerosis development can be attenuated.

## Results

### PIAS3 expression in atherosclerotic mouse aortas

**Figure 1:**
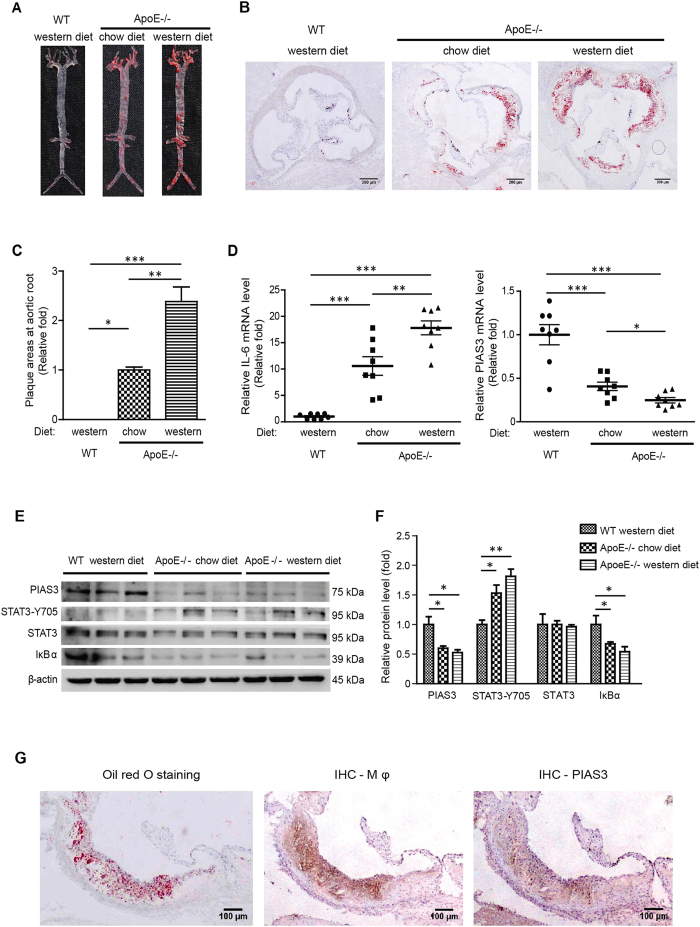
Atherosclerotic plaque formation is inversely correlated with PIAS3 expression in the aorta. (**A**) Representative images of Oil Red O-stained aortas. (**B**) Representative images showing atherosclerotic plaque formation at the aortic root (bar, 200 μm). (**C**) The relative sizes of the plaque areas at the aortic roots are expressed as fold changes relative to the sizes of the plaque areas of ApoE^−/−^ mice fed a chow diet (n = 8). Serial frozen sections of aortic roots were subjected to Oil Red O staining, and plaque areas were assessed. (**D**) IL-6 and PIAS3 gene expression levels in aortic tissue were determined by RT-qPCR (n = 8). (**E**) PIAS3, STAT3-Y705, STAT3 and IκBα protein expression levels in aortic tissue were determined by Western blotting. Full-length blots were presented in Supplementary Figure S1. (**F**) Densitometry analysis of the Western blotting results in panel E. The average protein levels after normalization with β-actin are expressed as fold changes relative to the protein levels in WT mice fed a western diet. (**G**) Representative images of PIAS3 protein detected in macrophages (Mφ) in atherosclerotic lesions. Serial frozen sections of aortic roots were subjected to immunohistochemical staining with Moma2 (for staining Mφ) and PIAS3 antibodies. Oil Red O staining was also performed to visualize the plaques (bar, 100 μm). *P < 0.05, **P < 0.01, ***P < 0.001.

JAK/STAT3 signalling is known to promote atherosclerosis development by facilitating vascular cell inflammation, proliferation, differentiation and migration^12^,^13^. PIAS3 is one of the main negative regulators of JAK/STAT3 signalling^19^. To assess PIAS3 expression and its correlation with atherosclerosis development, we fed wild-type (WT) mice and ApoE^−/−^ mice chow or western diets for 20 weeks. In contrast to WT mice, which exhibited no plaque formation, ApoE^−/−^ mice exhibited atherosclerotic plaques on the intima of their aortas (Fig. 1A). Moreover, ApoE^−/−^ mice fed western diets exhibited much higher plaque densities than ApoE^−/−^ mice fed chow diets. In addition, Oil Red O staining of frozen serial aortic root sections was performed. ApoE^−/−^ mice fed western diets exhibited an average plaque area that was 2.38-fold higher than that exhibited by ApoE^−/−^ mice fed chow diets (Fig. 1B,C).

IL-6 is a vascular inflammation indicator and activates JAK/STAT3 signalling to promote atherosclerosis development^9^. To understand the effects of PIAS3 on atherogenesis, we examined IL-6 and PIAS3 expression in atherosclerotic mouse aortas. ApoE^−/−^ mice fed chow diets exhibited significantly elevated IL-6 mRNA levels that were 10.6-fold higher than those exhibited by WT control mice, and ApoE^−/−^ mice fed western diets exhibited significantly elevated IL-6 mRNA levels that were 17.80-fold higher than those exhibited by WT control mice. In contrast, mice fed chow diets exhibited significantly reduced PIAS3 mRNA levels that were 0.41-fold those exhibited by WT control mice, and mice fed western diets exhibited significantly reduced PIAS3 mRNA levels that were 0.25-fold those exhibited by WT control mice (Fig. 1D). These results indicate that IL-6 levels were elevated in atherosclerotic lesions, whereas PIAS3 levels were reduced and were thus inversely correlated with atherosclerotic lesion formation.

To confirm the association between PIAS3 and atherosclerosis and assess JAK/STAT3 signalling activation and inflammation during atherosclerosis, we analysed PIAS3, STAT3-Y705, STAT3 and IκBα protein expression in aortas using Western blotting. ApoE^−/−^ mice exhibited significantly lower PIAS3 levels than WT control mice but exhibited increased phosphorylated STAT3 levels compared with WT control mice (Fig. 1E). In addition, IκBα protein levels were significantly decreased in ApoE^−/−^ mice, suggesting that NF-κB was activated. Densitometry analysis of our immunoblotting results confirmed the above findings and demonstrated that ApoE^−/−^ chow diet mice and ApoE^−/−^ western diet mice exhibited reduced PIAS3 levels that were 0.60-fold and 0.53-fold those exhibited by WT control mice, respectively (Fig. 1F). ApoE^−/−^ chow diet mice and ApoE^−/−^ western diet mice exhibited increased STAT3-Y705 levels that were 1.53-fold and 1.81-fold higher than those exhibited by WT control mice. Total STAT3 protein levels exhibited minimal changes among the three groups. ApoE^−/−^ mice fed chow and western diets exhibited decreased IκBα expression levels that were 0.67- and 0.54-fold those exhibited by WT control mice, respectively. These results indicate that PIAS3 expression levels were reduced in conjunction with atherosclerosis deterioration, while JAK/STAT3 signalling activation and the inflammatory response were upregulated.

Histological examinations of atherosclerotic plaques detected PIAS3 in the lesions and showed that PIAS3 was mainly localized in macrophages (Fig. 1G). As macrophages play a pivotal role in the inflammatory response during atherosclerosis^20^, a monocyte/macrophage cell line of mouse, RAW264.7, was used in subsequent experiments to determine the correlation between PIAS3 expression and atherosclerosis-related cellular responses.

### Reduction of PIAS3 expression in RAW264.7 cells stimulated by IL-6 or ox-LDL

**Figure 2:**
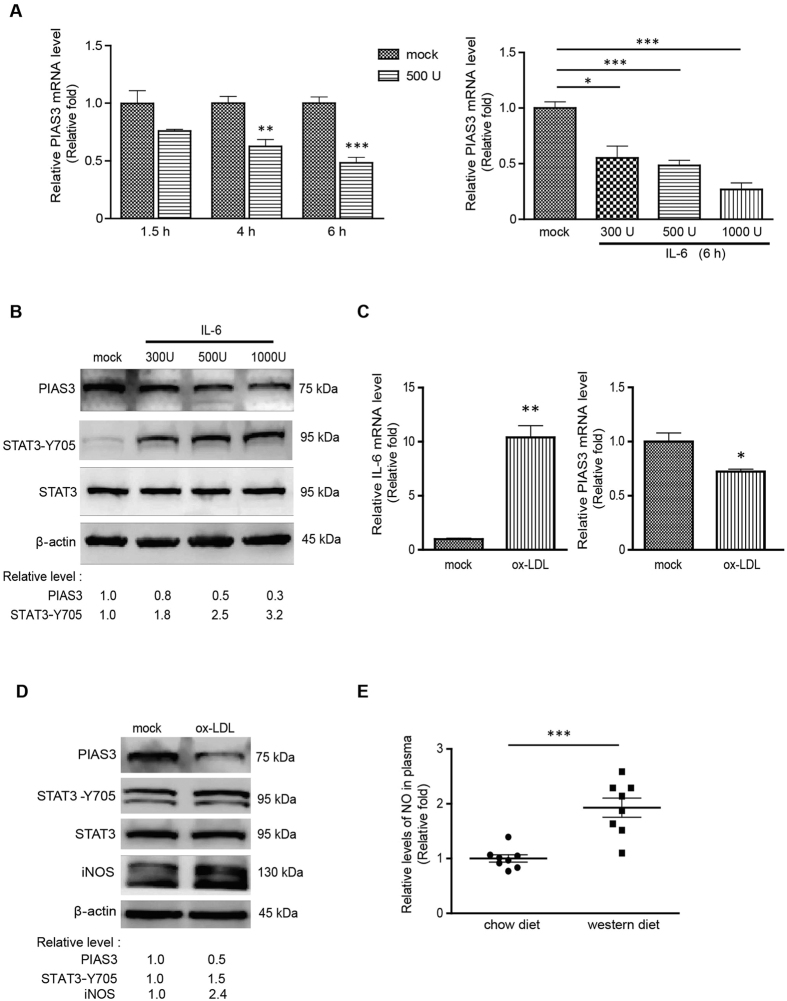
IL-6 or ox-LDL stimulation reduces PIAS3 expression in RAW264.7 cells. (**A**) IL-6 treatment leads to reductions in PIAS3 expression levels in a time- and dose-dependent manner, as determined via RT-qPCR. (**B**) Detection of PIAS3, STAT3-Y705 and STAT3 protein expression in IL-6-treated cells via Western blotting. Full-length blots were presented in Supplementary Figure S2. (**C**) Detection of IL-6 and PIAS3 expression in ox-LDL-stimulated cells via RT-qPCR. (**D**) Stimulation with ox-LDL reduces PIAS3 protein expression. PIAS3, STAT3-Y705, STAT3 and iNOS protein expression levels in ox-LDL-treated cells were detected via Western blotting. Full-length blots were presented in Supplementary Figure S3. (**E**) Relative levels of NO in the plasma of ApoE^−/−^ mice fed a 20-week chow or western diet. Fold changes in NO levels relative to NO levels in mice fed a chow diet are shown (n = 8). Regarding the RT-qPCR results, relative transcript levels are expressed as fold changes compared to transcript levels in mock-treated cells. Regarding the Western blotting results, the relative protein levels in treated cells compared with those in mock-treated cells are shown below the images. *P < 0.05, **P < 0.01, ***P < 0.001.

IL-6 is an inflammatory cytokine that is highly expressed during atherosclerosis. It can directly activate JAK/STAT3 signalling by interacting with the gp130 receptor. To determine PIAS3 expression levels in atherosclerotic inflammation, we used IL-6 to stimulate cells to mimic the atherosclerotic inflammatory response. The results showed that stimulating RAW264.7 cells with IL-6 resulted in reductions in PIAS3 mRNA levels in a time and dose-dependent manner (Fig. 2A). Cells treated with 500 U/ml IL-6 for 1.5, 4 and 6 hours exhibited reduced PIAS3 mRNA expression levels that were 0.76-, 0.63- and 0.48-fold those exhibied by mock-treated cells, respectively. Cells treated with 300, 500 and 1000 U/ml IL-6 for 6 hours exhibited reduced PIAS3 mRNA levels that were 0.55-, 0.48- and 0.27-fold those exhibited by mock-treated cells, respectively. On protein level, along with the incremental increase of IL-6, PIAS3 protein levels also dropped gradually, whereas STAT3-Y705 levels significantly increased (Fig. 2B). Densitometry analysis showed that, compared to the mock-treated cells, the PIAS3 protein levels in the cells treated by IL-6 at 300 and 1000 U/mL were reduced to 0.8 and 0.3-fold, respectively, whereas the STAT3-Y705 levels were elevated to 1.8 and 3.2-fold, respectively. These results indicate that IL-6-induced activation of JAK/STAT3 signalling led to reductions in PIAS3 expression.

High levels of ox-LDL that are commonly detected in the plasma of patients with coronary artery disease are a risk factor of atherosclerosis^21^. Therefore, we directly stimulated RAW264.7 cells with ox-LDL to examine the changes in PIAS3 expression induced by this treatment. Treating RAW264.7 cells with 100 μg/ml ox-LDL for 24 hours led to a significant 10.4-fold increase in IL-6 mRNA expression compared with mock-treated cells and a decrease in PIAS3 mRNA expression to levels that were 0.72-fold those of mock-treated cells (Fig. 2C). Furthermore, the PIAS3 protein expression levels exhibited by ox-LDL-treated cells were 0.5-fold those exhibited by mock-treated cells, whereas the STAT3-Y705 protein levels exhibited by these cells were 1.5-fold higher than those exhibited by mock-treated cells (Fig. 2D).

It has been reported that nitric oxide (NO) promotes PIAS3 degradation by facilitating interactions between PIAS3 and ubiquitin E3 ligase^22^. Here, we detected inducible nitric oxide synthase (iNOS) expression in ox-LDL-treated cells. Compared to the mock-treated cells, the iNOS level in the ox-LDL stimulated cells showed a 2.4-fold upregulation (Fig. 2D). Plasma NO levels in ApoE^−/−^ mice fed western diets were 1.93-fold higher than those in ApoE^−/−^ mice fed chow diets (Fig. 2E), which may partially explain the abovementioned reductions in PIAS3 expression levels.

### PIAS3 overexpression suppresses ox-LDL-induced inflammatory responses

**Figure 3:**
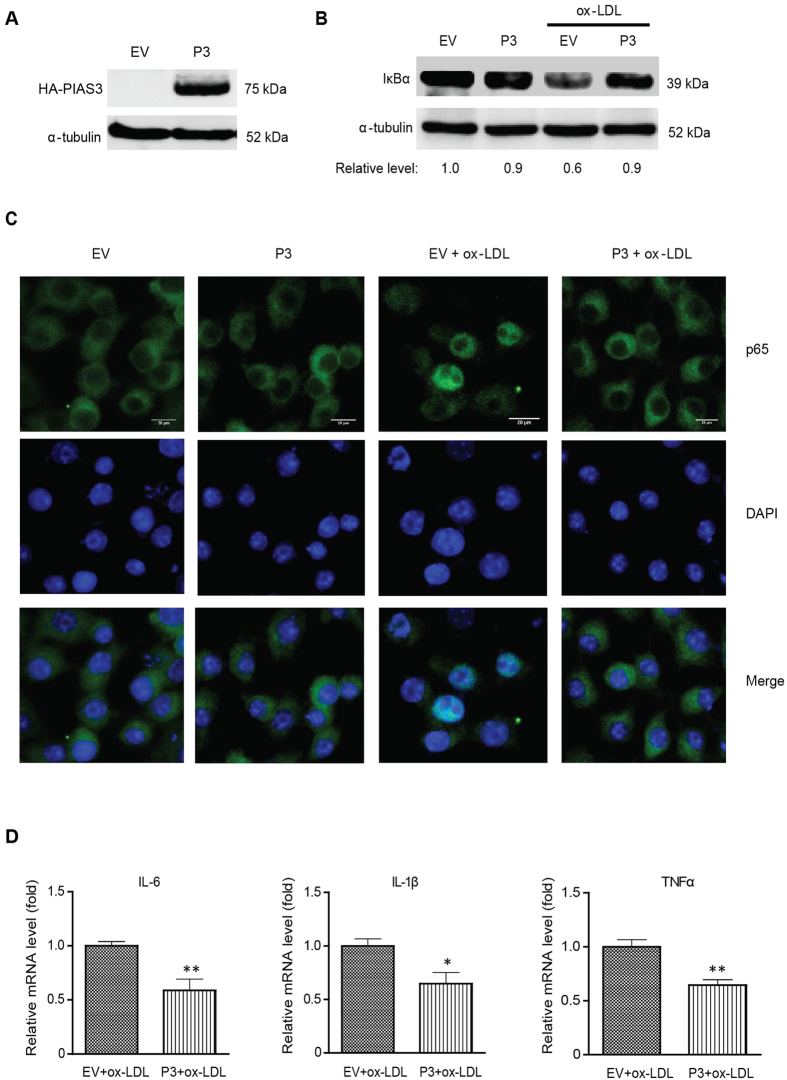
PIAS3 overexpression attenuates ox-LDL-induced inflammatory responses in RAW.264.7 cells. (**A**) Detection of exogenous PIAS3 in the cells via immunoblotting. The cells were transfected with empty vector (EV) or the pCAGEN-HA-PIAS3 plasmid (P3) and then harvested for Western blotting with anti-HA and α-tubulin antibodies. Full-length blots were presented in Supplementary Figure S4. (**B**) PIAS3 inhibits IκBα degradation in ox-LDL treated-cells. Relative fold changes in IκBα protein levels are shown below the images. Full-length blots were presented in Supplementary Figure S5. (**C**) PIAS3 blocks p65 nuclear translocation in ox-LDL-treated cells. (bar, 20 μm). (**D**) PIAS3 reduces inflammatory cytokine expression in ox-LDL treated cells. Relative levels of IL-6, IL-1β and TNF-α transcript detected by RT-qPCR are expressed as fold changes compared to transcript levels in EV-transfected cells. *P < 0.05, **P < 0.01.

To investigate the role of PIAS3 in atherosclerotic inflammation further, we cloned PIAS3 and used the resulting plasmid, pCAGEN-HA-PIAS3, to induce transient PIAS3 expression. PIAS3 expression in cells transfected with the recombinant pCAGEN-HA-PIAS3 plasmid was confirmed by Western blotting (Fig. 3A). In cells transfected with empty vector (EV), ox-LDL administration induced reductions in endogenous IκBα protein expression to levels that were 0.6-fold those observed in mock-treated cells (Fig. 3B). In contrast, IκBα levels in PIAS3-overexpressing and ox-LDL-treated cells were much higher than those in cells transfected with EV (Fig. 3B), indicating that PIAS3 blocks ox-LDL-mediated IκBα degradation.

During NF-κB activation, IκBα degradation is followed by p65 nuclear translocation. We therefore subsequently analysed the distribution of p65 in ox-LDL-treated PIAS3-overexpressing cells using immunofluorescence assay (IFA). The results indicated that upon ox-LDL treatment, p65 mainly relocated to the nucleus in cells transfected with EV, whereas it remained in the cytoplasm in cells transfected with PIAS3 (Fig. 3C). These results indicate that PIAS3 overexpression led to the inhibition of ox-LDL-induced p65 nuclear translocation. In addition, in ox-LDL-stimulated cells, PIAS3 overexpression led to significant reductions in the mRNA expression of the proinflammatory cytokines IL-6, IL-1β and tumour necrosis factor-α (TNF-α) to levels that were 0.58-, 0.64-, and 0.64-fold those observed in EV-transfected cells, respectively (Fig. 3D).

### PIAS3 inhibits ox-LDL-induced lipid accumulation in RAW264.7 cells

**Figure 4:**
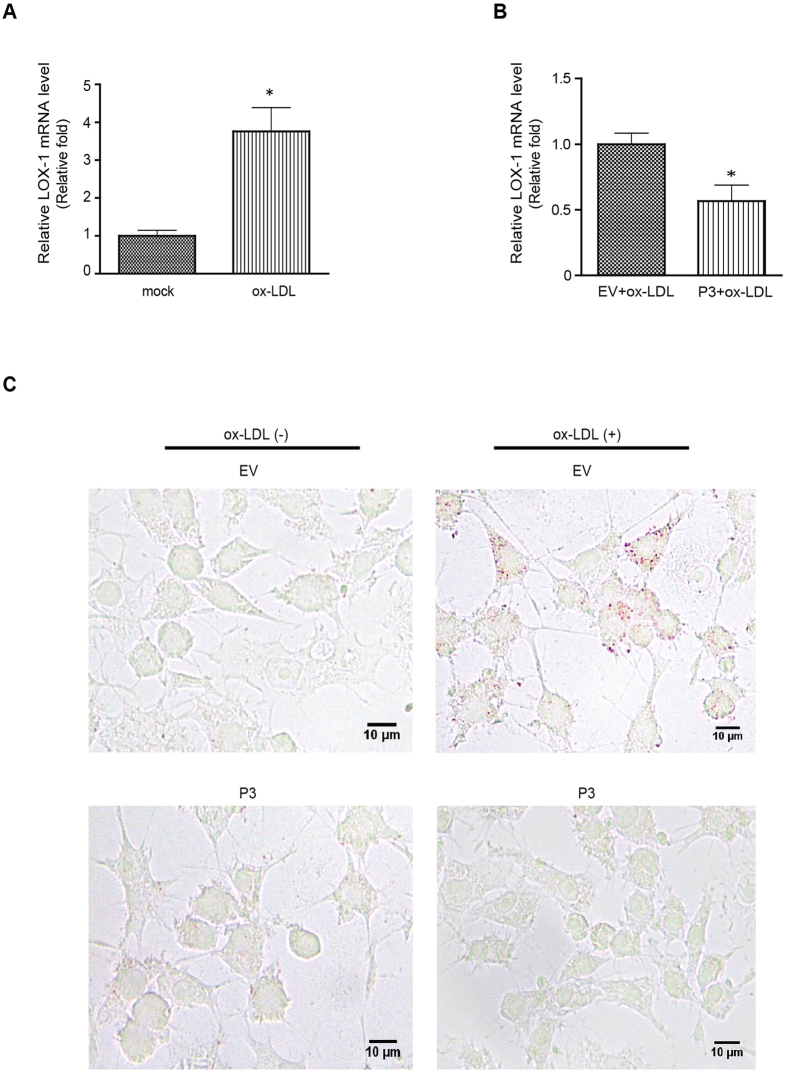
PIAS3 impairs ox-LDL-induced lipid accumulation in RAW264.7 cells. (**A**) Ox-LDL stimulation results in increased LOX-1 expression. Relative levels of LOX-1 transcripts were determined by RT-qPCR and are expressed as fold changes compared to transcript levels in mock-treated cells. (**B**) PIAS3 suppresses ox-LDL-induced LOX-1 expression. LOX-1 transcript levels were detected by RT-qPCR and expressed as fold changes compared to transcript levels in EV-transfected cells. (**C**) PIAS3 inhibits ox-LDL-induced lipid accumulation in RAW264.7 cells, as determined via Oil Red O staining. The red dots represent lipids accumulated in the cells (bar, 10 μm). *P < 0.05.

Macrophages internalize ox-LDL via several scavenger receptors, including lectin-like ox-LDL receptor-1 (LOX-1), resulting in lipid accumulation and the consequent transformation of macrophages into foam cells, which is the typical pathological process that occurs during atherosclerosis^21^. Basal LOX-1 expression levels are very low and are generally upregulated by several proinflammatory and proatherogenic stimuli^23^. In human carotid atherosclerotic plaques, LOX-1 is highly expressed in macrophages^24^. Here, we found that LOX-1 mRNA expression in RAW264.7 cells increased to a level that was 3.76-fold that observed in mock-treated cells in response to ox-LDL treatment for 24 hours (Fig. 4A). However, PIAS3 overexpression suppressed this ox-LDL-induced LOX-1 upregulation (n-fold of PIAS3-transfected versus EV-transfected cells ≈0.56) (Fig. 4B). Oil Red O staining of ox-LDL treated cells showed that ox-LDL stimulation promoted lipid accumulation in the cytoplasm of macrophages, while PIAS3 blocked ox-LDL-induced lipid uptake (Fig. 4C). Hence, PIAS3 is an important factor to interfere with atherogenesis by attenuating lipid accumulation and hampering foam cell formation.

### PIAS3 suppresses ox-LDL-induced VSMC proliferation by attenuating reactive oxygen species generation

**Figure 5:**
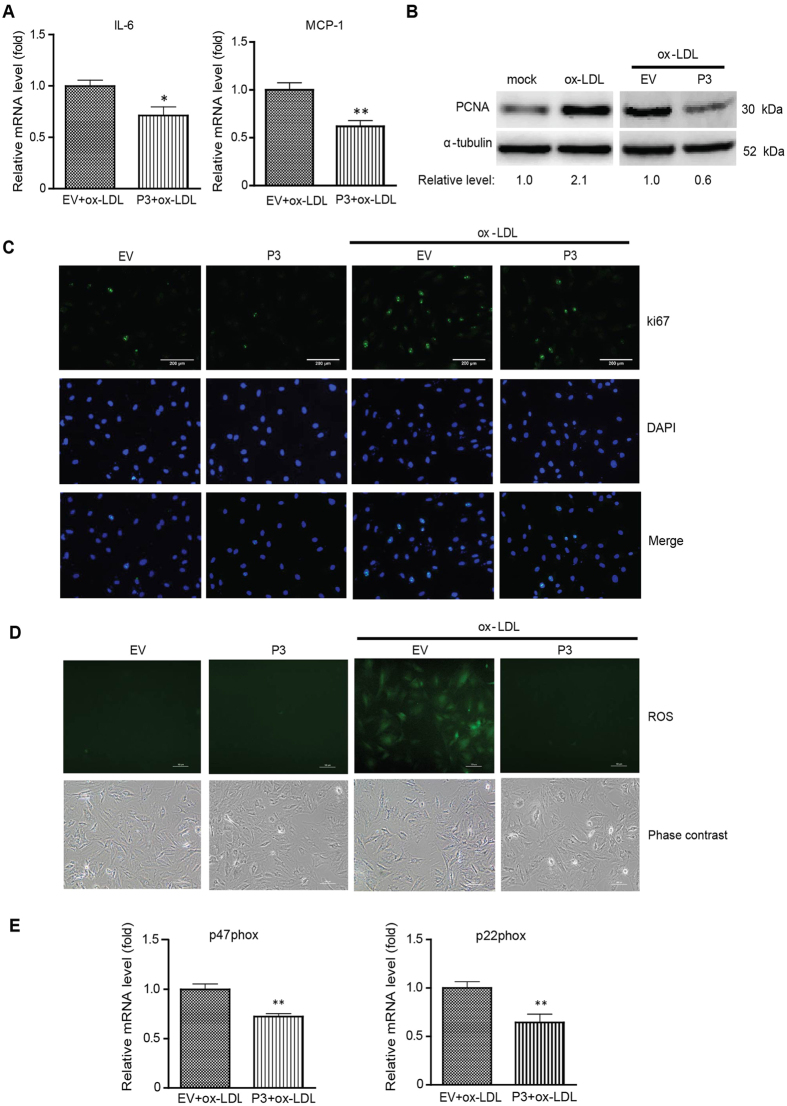
PIAS3 inhibits VSMC proliferation by attenuating reactive oxygen species generation. (**A**) PIAS3 downregulates inflammatory cytokine expression in ox-LDL-treated VSMCs. The relative mRNA levels of IL-6 and MCP-1, as determined via RT-qPCR, are expressed as fold changes compared with the corresponding mRNA levels in EV-transfected cells. (**B**) PIAS3 suppresses the elevation of PCNA expression in ox-LDL-treated VSMCs. Relative fold changes in PCNA protein levels are shown below the images. Full-length blots were presented in Supplementary Figure S6. (**C**) PIAS3 inhibits ox-LDL-induced VSMC proliferation, as determined via immunofluorescence assay using antibody against ki67 (bar, 200 μm). (**D**) PIAS3 attenuates ox-LDL-induced ROS production in VSMCs (bar, 100 μm). (**E**) PIAS3 reduces NADPH oxidase subunit expression in ox-LDL-treated cells. The relative levels of the p47phox and p22phox transcripts, as determined via RT-qPCR, are expressed as fold changes compared to the levels of these transcripts in EV-transfected cells. *P < 0.05, **P < 0.01.

In addition to macrophages, VSMCs are also involved in atherosclerosis development. VSMC proliferation and migration are associated with plaque formation and stabilization. VSMCs that have migrated to the intima are capable of producing cytokines that attract and activate leukocytes^25^. Our results indicated that PIAS3 suppressed IL-6 and monocyte chemotactic protein-1 (MCP-1) expression in ox-LDL-treated VSMCs. PIAS3 reduced IL-6 and MCP-1 expression to levels that were 0.71-fold and 0.61-fold those observed in EV-transfected cells, respectively (Fig. 5A). Proliferating cell nuclear antigen (PCNA) and ki67 are common proliferation markers^26^. Our Western blotting results demonstrated that upon ox-LDL stimulation, VSMCs exhibited a 2.1-fold increase in PCNA expression, whereas PIAS3-overexpressing ox-LDL-treated VSMCs significantly reduced PCNA expression to levels that were 0.6-fold those observed in EV-transfected ox-LDL-treated VSMCs (Fig. 5B). Similarly, our IFA results indicated that upon ox-LDL treatment, EV-transfected VSMCs exhibited increases in ki67 expression, whereas PIAS3-overexpressing ox-LDL-treated VSMCs exhibited much lower ki67 expression than EV-transfected ox-LDL-treated VSMCs (Fig. 5C). These results indicate that PIAS3 inhibits ox-LDL-induced VSMC proliferation.

Reactive oxygen species (ROS) are a class of oxygen-derived molecules that play prominent roles in the cardiovascular system. During atherosclerosis, ROS mediate pathophysiological processes, including VSMC proliferation and migration^27^. In addition, a relationship exists between ROS production and JAK/STAT3 singling activation^7^,^28^. Based on the above results, we speculated PIAS3 reduces ROS production by impeding STAT3-mediated signalling and consequently inhibiting ox-LDL-induced VSMC proliferation. Here, we detected ROS production by adding a probe, named 2′,7′-dichlorofluorescein diacetate (DCFH-DA), into ox-LDL-treated VSMCs. Large amounts of ROS were generated in EV-transfected cells after ox-LDL treatment; however, no ROS were observed in PIAS3-transfected cells upon ox-LDL stimulation (Fig. 5D). NADPH oxidases (Nox) are the enzymes mainly responsible for ROS generation. Compared with EV-transfected cells, PIAS3-overexpressing cells exhibited significantly lower mRNA expression levels of the Nox subunits p22phox and p47phox. Specifically, these levels were 0.64-fold and 0.72-fold those exhibited by EV-transfected cells, respectively (Fig. 5E). These results indicate that PIAS3 is capable of inhibiting ox-LDL-induced ROS production and may also shed light on the mechanism underlying PIAS3-mediated VSMC proliferation inhibition.

## Discussion

**Figure 6:**
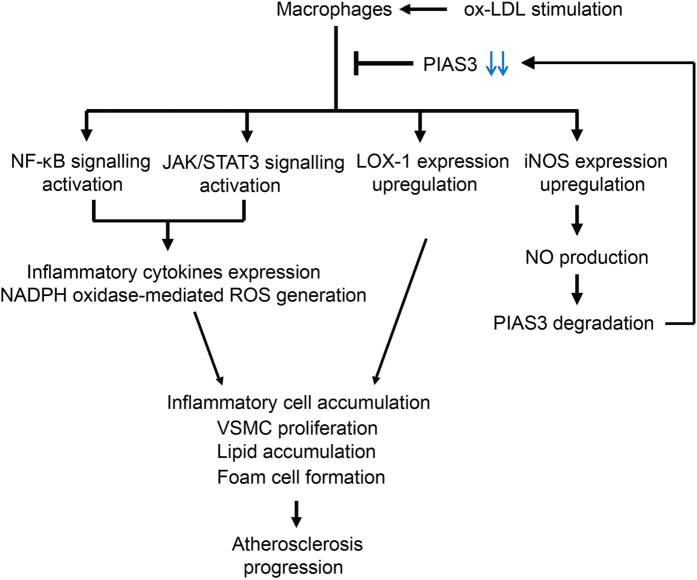
Proposed mechanism underlying ox-LDL-induced atherosclerosis progression. NF-κB and JAK/STAT3 signalling were activated in ox-LDL-stimulated macrophages, thereby increasing inflammatory cytokines expression and ROS generation, effects that led to inflammatory cell accumulation and VSMC proliferation. LOX-1 and iNOS expression were also upregulated in the stimulated cells, which facilitated foam cell formation and NO production, respectively. NO triggered PIAS3 degradation, thereby attenuating the inhibition effect of PIAS3 on atherosclerosis progression. (ox-LDL, oxidized low-density lipoprotein; NF-κB, nuclear factor-κB; JAK/STAT3, janus kinase/signal transducer and activator of transcription 3; LOX-1, lectin-like ox-LDL receptor-1; iNOS, inducible nitric oxide synthase; NADPH oxidase, nicotinamide adenine dinucleotide phosphate-oxidase; ROS, reactive oxygen species; NO, nitric oxide; VSMC, vascular smooth muscle cell; PIAS3, protein inhibitor of activated STAT3).

Atherosclerosis is a chronic multi-factorial disease characterized by prominent inflammation-induced injuries to the intima of arteries^20^. The JAK/STAT signalling pathway plays an important role in atherosclerosis initiation and progression^9^,^10^. We found that PIAS3, a key negative regulator of JAK/STAT3 signalling, was downregulated in atherosclerotic aortas in mice and that its expression may be inversely correlated with atherosclerosis progression. Our results indicate that PIAS3 suppressed ox-LDL-induced inflammation, lipid accumulation in macrophages and VSMC proliferation, which are that main cellular responses that occur during atherosclerosis. The possible mechanisms underlying ox-LDL-induced atherosclerosis development in ApoE^−/−^ mice are schematically represented in Fig. 6.

It is known that JAK/STAT signalling and the inflammatory response contribute significantly to atherosclerosis progression^7^,^16^,^29^,^30^,^31^. Our results indicated that atherosclerotic mouse aortas exhibited elevated STAT3 phosphorylation levels and reduced IκBα levels, suggesting that the JAK/STAT3 signalling pathway and NF-κB pathway are activated during atherosclerosis. Interestingly, PIAS3 expression levels decreased significantly in conjunction with atherosclerotic deterioration, suggesting that PIAS3 levels in aortas are inversely correlated with atherosclerosis development.

To explore the correlation between PIAS3 and atherosclerosis further, we conducted experiments with proinflammatory cytokine IL-6, which is highly expressed in atherosclerotic aortas and can directly activate gp130/JAK/STAT3 signalling to exacerbate atherosclerosis by promoting cell inflammation, proliferation, differentiation and migration^9^,^10^. We noticed that PIAS3 expression levels decreased along with JAK/STAT3 signalling activation in IL-6-stimulated cells in a time and dose-dependent manner. In addition, we observed that the atherogenic stimulus ox-LDL is capable of inducing increases in IL-6 expression and reductions in PIAS3 expression. These results are consistent with those of our mouse experiments and support the hypothesis that PIAS3 expression is inversely correlated with atherosclerosis development.

The mechanisms underlying the abovementioned decreases in PIAS3 expression were subsequently evaluated. It has been reported that NO triggers PIAS3 S-nitrosation, which promotes PIAS3 degradation by facilitating its interaction with tripartite motif-containing 32 (TRIM32), an E3 ubiquitin ligase^22^. This finding suggests that NO stimulates PIAS3 degradation. Our results indicated that plasma NO levels increased significantly in conjunction with increases in atherosclerosis severity. Furthermore, iNOS expression levels were markedly upregulated in ox-LDL treated macrophages, whereas PIAS3 expression levels were decreased in these macrophages. NO synthesis upregulation may therefore be one of the mechanisms underlying the abovementioned reductions in PIAS3 expression.

It is known that in addition to PIAS, the SOCS protein family is also the regulator of JAK/STAT signalling^14^,^19^. Previous studies have shown that SOCS1 and SOCS3 are closely correlated with atherosclerosis progression^15^,^16^,^17^. However, they appear to have different expression patterns and play opposing roles during atherogenesis^17^,^32^,^33^. In ApoE^−/−^ mouse aortas, SOCS1 expression levels first increased and then decreased during the 22-week feeding period, whereas SOCS3 expression levels increased continuously along with the elongation of feeding^17^. Interestingly, we found that PIAS3 expression, unlike SOCS1 expression, exhibits significant declines, even during the early stages of atherosclerosis. During the fourth week of western diet feeding, PIAS3 mRNA levels in ApoE^−/−^ mice had already decreased. Compared to WT mice, ApoE^−/−^ mice exhibited decreased PIAS3 mRNA expression in aortas. Specifically, their PIAS3 mRNA levels were 0.73-fold those exhibited by WT mice. However, ApoE^−/−^ mice exhibited increased IL-6 mRNA levels that were 2.86-fold those exhibited by WT mice (data not shown). Atherosclerosis progression is associated with PIAS3 downregulation, suggesting that PIAS3 plays a crucial role in attenuating atherosclerosis development. Hence, we performed a series of experiments using cultured cells to determine the effects of PIAS3 on atherosclerosis and found that PIAS3 overexpression suppressed ox-LDL-induced inflammation, lipid accumulation and VSMC proliferation.

Excessive inflammation is a typical feature of atherosclerosis^34^. Activation of the nuclear transcription factor NF-κB leads to inflammatory cytokine expression. IκBα degradation is a critical step in NF-κB activation and nuclear translocation. Our results indicate that IκBα degradation and NF-κB subunit p65 nuclear translocation in ox-LDL treated cells, findings consistent with those of previous reports showing that ox-LDL induces inflammatory responses^35^,^36^. Interestingly, we found that PIAS3 suppressed ox-LDL-induced IκBα degradation, p65 nuclear translocation and inflammatory cytokine expression, suggesting that PIAS3 attenuates inflammation by inhibiting NF-κB activation. This result can be explained by the findings of previous studies showing that PIAS3 directly associates with p65 to interfere with p65-CBP coactivator binding and mediate p65 SUMOylation^37^,^38^.

Lipid-laden macrophage accumulation is a hallmark of atherosclerosis. Intimal macrophages ingest ox-LDL via several scavenger receptors, such as SR-AI/II, CD36 and LOX-1, resulting in the transformation of macrophages into foam cells^39^. It has been reported that in inflamed microenvironments exhibiting high TNF-α and transforming growth factor-β expression levels, LOX-1 expression levels were upregulated, whereas those of other SRs (SR-AI/II and CD36) were downregulated, suggesting that LOX-1 plays a significant role in ox-LDL uptake by macrophages^40^,^41^,^42^. We found that ox-LDL significantly upregulates LOX-1 expression in RAW264.7 cells and that large amounts of lipid droplets accumulate in ox-LDL-stimulated cells. However, PIAS3 suppresses LOX-1 expression and lipid accumulation. PIAS3 is capable of decreasing TNF-α and LOX-1 expression, which may be the mechanism underlying its inhibition of lipid accumulation in PIAS3-overexpressing cells.

Abnormal VSMC proliferation is a major contributor to atherosclerosis development^25^,^43^,^44^. ROS are essential for maintaining vascular structure and regulating metabolic process, such as cell growth, proliferation, migration, etc. However, ROS overproduction may result in inflammation and metabolic dysfunction, thereby promoting cardiovascular disorders^27^. NADPH oxidases (Nox), a class of hetero-oligomeric enzymes, are mainly responsible for ROS generation. In response to proatherogenic stimuli, Nox activity levels are highly elevated, resulting in excessive ROS production and atherogenesis acceleration. Previous studies have demonstrated ROS are capable of activating JAK/STAT signalling and that Nox inhibition can effectively prevent JAK2, STAT1 and STAT3 phosphorylation^7^,^45^. JAK/STAT pathway inhibition significantly diminishes Nox activity and expression and subsequently hampers ROS production^28^. All of these data suggest that a relationship exists between JAK/STAT signalling and ROS generation. Our results indicated that PIAS3 attenuated ox-LDL-induced VSMC proliferation. As ROS is a key inducer of cell proliferation, and ROS production is influenced by JAK/STAT signalling, we hypothesized that PIAS3 attenuates VSMC proliferation by abating ROS generation. We noticed that PIAS3 reduced ROS production and Nox subunit expression in ox-LDL-treated VSMCs. PIAS3-mediated inhibition of ROS generation may be caused by PIAS3-mediated inhibition of JAK/STAT3 signalling. Additional studies are needed to delineate the detail mechanism underlying this process.

In conclusion, we determined that PIAS3 expression is inversely correlated with atherosclerosis development. Atherogenic stimuli, such as IL-6 and ox-LDL, directly reduce PIAS3 expression levels, an effect that may be due to NO synthesis upregulation. PIAS3 suppresses ox-LDL-induced inflammatory responses, lipid accumulation and VSMC proliferation. Our data suggest that PIAS3 is a critical repressor of atherosclerosis progression. Our findings have contributed to our understanding on the pathogenesis of atherosclerosis and have provided us with a potential target through which we can attenuate atherosclerosis development. PIAS3 upregulation should be explored as a potential strategy for treating atherosclerosis.

## Materials and Methods

### Reagents and plasmids

To analyse inflammatory responses during atherosclerosis in cultured cells, murine IL-6 (Cat. No. 216-16, Peprotech Inc., Rocky Hill, NJ, USA) and human ox-LDL (Cat. No. YB-002, Yiyuan Biotechnologies, Guangzhou, China) were used at a final concentration of 500 U/ml and 100 μg/ml, respectively (unless stated differently in the Results and Figure Legends). These cells were harvested for further analysis at the time points indicated in the Results and Figure Legends.

Gene cloning was performed as previously described using a pCAGEN-HA vector^46^. The murine PIAS3 gene sequence was amplified using the primers mPIAS3-clone-F and m-PIAS3-clone-R (Supplementary Table S1) and was then cloned into the pCAGEN-HA vector. The resulting recombinant plasmid, pCAGEN-HA-PIAS3, was confirmed via restriction enzyme digestion and DNA sequencing.

### Animal study

The animal experimental protocol was approved by the Laboratory Animal Administration Committee of Xi’an Jiaotong University and was carried out in accordance with the Guidelines for Animal Experimentation of Xi’an Jiaotong University and the Guide for the Care and Use of Laboratory Animals published by the US National Institutes of Health (NIH Publication NO. 85-23, revised 2011). A total of 22 ApoE^−/−^ mice (8-week-old males, C57BL/6J background) were randomly separated into two groups (n = 11 per group) and were fed a chow diet or western diet (containing 0.15% cholesterol and 21% fat, wt/wt) for 20 weeks, respectively. Meanwhile, 11 wild-type mice (C57BL/6J, 8 week-old males) were fed a western diet for 20 weeks and served as a control group. The mice were euthanized humanely via pentobarbital overdoses administered via intraperitoneal injections. The aortas from some of the euthanized mice (n = 3 per group) were isolated for Oil Red O staining. The aortas from the remaining euthanized mice (n = 8 per group) were divided into two portions as follows: the upper (aortic root) portion was used for histologic analysis, and the abdominal/thoracic aorta was used for mRNA and protein expression analyses.

### Cells

RAW264.7 cells were grown in Dulbecco’s Modified Eagle’s Medium (DMEM) supplemented with 10% fetal bovine serum (FBS). Primary VSMCs were isolated from rat thoracic aortas as described previously^47^. Briefly, young male Sprague-Dawley rats (~200 grams) were euthanized humanely via pentobarbital overdoses administered via intraperitoneal injections. The thoracic aorta was isolated, opened longitudinally, and stripped of its intima and adventitia. The smooth muscle tissue was minced and placed in culture plates containing DMEM with 20% FBS supplemented with penicillin (100 U/ml) and streptomycin (100 μg/ml). The VSMCs derived from these explants were passaged at confluence and subsequently cultured in DMEM with 10% FBS. Characterization of the cell was performed via immunofluorescence assay using anti-α smooth muscle actin antibody (Cat. No. ab202510, Abcam, Cambridge, UK). VSMCs from the third through the fifth passages were used, and at least six batches of VSMCs were studied in each experiment. Before being treated with IL-6 or ox-LDL, the cells were made quiescent by 24 h of incubation in plain culture medium without FBS.

### Histology and immunohistochemistry

The mouse aortic roots were processed into serial frozen sections of 7 μm thickness (covering approximately 672 μm from the valve leaflets). The sections were stained by 0.5% Oil Red O for detecting neutral lipids in plaques. In addition, for detection of macrophages and PIAS3 in atherosclerotic plaques at aortic root, the sections were immunohistochemically stained with antibodies against either Moma2 (Cat. No. ab33451, Abcam) or PIAS3 (Cat. No. PA5-20953, Thermo Fisher Scientific, Waltham, MA, USA) as described previously^48^. Histological features of the atherosclerotic lesions were evaluated by an independent pathologist and then, all images were recorded via NIS-Elements F3.2 software under a Nikon ECLIPSE 80i microscope equipped with a Nikon DS-Ri1 camera. Positive areas of Oil Red O staining in the lesions were quantified blindly by a laboratory member who did not know the group information using WinROOF V6.5 analysis software as described previously^48^. Eight cross-sections of vessels in each aorta were analysed and averaged for each animal. Representative images of immunohistochemically staining were used to show PIAS3 protein and macrophages (Moma2 protein) in atherosclerotic lesions.

### Western blotting

Proteins in test samples were separated by sodium dodecyl sulfate-polyacrylamide gel electrophoresis (SDS-PAGE) and analysed by Western blotting, as described previously^46^. The separated proteins were subsequently transferred onto PVDF membranes and probed with antibodies against phosphorylated STAT3 at tyrosine-705 (STAT3-Y705) (Cat. No. 9145, Cell Signaling Technology, Danvers, MA, USA), STAT3 (Cat. No. 4904, Cell Signaling), PIAS3 (Thermo Fisher Scientific), IκBα (Cat. No. 4812, Cell Signaling), p65 (Cat. No. 8242, Abcam), iNOS (Cat. No. ab3523, Abcam), PCNA (Cat. No. ab15497, Abcam), β-actin (Cat. No. ab8227, Abcam), α-tubulin (Cat. No. ab15246, Abcam), and haemagglutinin (HA) (Cat. No. ab20084, Abcam). The membrane-bound antibodies were detected with secondary antibodies conjugated with horseradish peroxidase (Cat. No. 31460, Thermo Fisher Scientific) before being detected with a chemiluminescence substrate. The luminescence signals were recorded digitally using a Chemi-Doc XRS imaging system (Bio-Rad Laboratories, Hercules, CA, USA). Digital image acquisition and analysis were conducted using the Quantity One software (Bio-Rad). Protein levels quantitated via western blotting were normalized by loading internal controls.

### RNA isolation and real-time PCR

Total RNA was isolated from the cells and tissues using TRIzol reagent (Cat. No. 15596-018, Invitrogen, Grand Island, NY, USA), in accordance with the manufacturer’s instructions. Reverse transcription and real-time quantitative PCR (RT-qPCR) were conducted as previously described^46^. The real-time PCR primers for PIAS3, IL-6, IL-1β, TNF-α, MCP-1, LOX-1, p22phox, and p47phox are listed in Supplementary Table S1. The transcripts of the house-keeping gene ribosomal protein L32 (RPL32) were also amplified and used to normalize the total input RNA. Transcript levels were quantified using the 2^−ΔΔCT^ threshold cycle method^49^ and were shown as fold changes relative to the transcript levels of the control.

### Immunofluorescence assay (IFA)

The cells were seeded into culture plate wells with coverslips added and incubated overnight before being transfected and treated with ox-LDL. IFA was performed as described previously using antibodies against p65 or ki67 (Cat. No. ab66155, Abcam)^46^. The coverslips were mounted onto the slides using Fluoromount-G clear mounting medium containing 4′,6′-diamidino-2-phenylindole (DAPI) (Cat. No. 0100-20, Southern Biotech, Birmingham, AL, USA). The fluorescence signals were observed via fluorescence microscopy (Nikon ECLIPSE Ti), and images were taken using NIS-Elements F software (Nikon).

### Nitric oxide (NO) detection

Blood samples treated with EDTA were collected from the euthanized mice. These samples were then centrifuged at 3,000 rpm for 15 min, after which the plasma was collected for NO metabolite detection, which was performed using a Nitrite/Nitrate Assay Kit (Cat. No. 23479, Sigma-Aldrich Corp, St. Louis, MO, USA), according to the manufacturer’s instructions^50^,^51^. Each sample was measured in triplicate. In each group, we measured 8 samples and the results were expressed as the mean ± standard error. The reproducibility of NO detection had been confirmed via performing a pilot study using the same samples measured by different members.

### Reactive oxygen species (ROS) detection

2′,7′-dichlorofluorescein diacetate (DCFH-DA) (Cat. No. D6883, Sigma), a cell-permeable non-fluorescent probe, was used to measure hydroxyl, peroxyl and other ROS activity within the cells^50^,^52^. DCFH-DA is de-esterified intracellularly and turns to highly fluorescent 2′,7′-dichlorofluorescein upon oxidation. The fluorescence signal can be easily detected by fluorescence microscopy. We performed a pilot study to confirm the reproducibility of the assay. DCFH-DA was added to the cultured cells at a final concentration of 10 μM for 30 min of incubation. The cells were then rinsed three times with plain culture medium without FBS before being evaluated by fluorescence microscopy (Nikon ECLIPSE Ti). The NIS-Elements F software (Nikon) was used to acquire images. The ROS detection assay were performed six times and the representative images were shown in the result.

### Statistical analysis

Data are expressed as the mean ± standard error. Student’s *t-*tests (for comparisons between two groups) or one-way analysis of variance (ANOVA) (for comparisons of ≥3 groups) followed by Tukey’s post hoc test was used for the statistical analyses. SPSS software version 17.0 (SPSS, Chicago, IL) was used for all data analyses. A value of P < 0.05 was considered statically significant.

## Additional Information

**How to cite this article**: Wang, R. *et al*. Protein Inhibitor of Activated STAT3 Suppresses Oxidized LDL-induced Cell Responses during Atherosclerosis in Apolipoprotein E-deficient Mice. *Sci. Rep.* **6**, 36790; doi: 10.1038/srep36790 (2016).

**Publisher’s note**: Springer Nature remains neutral with regard to jurisdictional claims in published maps and institutional affiliations.

## Supplementary Material

Supplementary Information
