# Supplementary material for: Protein Inhibitor of Activated STAT3 Suppresses Oxidized LDL-induced Cell Responses during Atherosclerosis in Apolipoprotein E-deficient Mice
Source: Sci Rep. 2016 Nov 15;6:36790. doi: 10.1038/srep36790 (PMC5109228; doi:10.1038/srep36790)
Supplement: Supplementary Information [file srep36790-s1.pdf]

Protein Inhibitor of Activated STAT3 Suppresses Oxidized LDL-induced Cell Responses during Atherosclerosis in Apolipoprotein E-deficient Mice

Rong Wang, Yanjin Zhang, Liran Xu, Yan Lin, Xiaofeng Yang, Liang Bai, Yulong Chen, Sihai Zhao, Jianglin Fan, Xianwu Cheng and Enqi Liu

Supplementary Table S1. List of primers for gene cloning or real-time PCR.

**Primer ***	**Sequences (5'-3')**
mPIAS3-clone-F	CGTGATGAGTTTCCGAGTGTCTG
mPIAS3-clone-R	CGCTCGAGTCAGTCCAAGGAAATG
mrRPL32-F	GAGGCATTGACAACAGGGTG
mRPL32-R	CTGACATTGTGGACCAGGAAC
rRPL32-R	CTTGACATTGTGGACCAGAAAC
mPIAS3-F	AGAAGGAGGCATCAGAGGTTTG
rPIAS3-F	AAGGAGGCATCCGAGGTTTG
mrPIAS3-R	GCTTTCGATGGTCAAGTCAATG
mrIL6-F	CCACTTCACAAGTCGGAGGCTTA
mrIL6-R	CCAGTTTGGTAGCATCCATCATTTC
mIL1β-F	CGTGGACCTTCCAGGATGAG
mIL1β-R	CATCTCGGAGCCTGTAGTGC
mTNFα-F	TGAGCACAGAAAGCATGATCC
mTNFα-R	GCCATTTGGGAACTTCTCATC
mLOX1-F	TTCAGAACCTCCAAGAAGCC
mLOX1-R	AGCTTCAAGGTGAGGGTGTC
rMCP1-F	AGATGCAGTTAACGCCCCAC
rMCP1-R	CCCATTCCTTCTTGGGGTCA
rp22phox-F	CCATCAAGCAGCCACCTAC
rp22phox-R	CATCTGTCACTGGAATTGGG
rp47phox-F	GAGACATACCTGACGGCCAAAGA
rp47phox-R	AGTCAGCGATGGCCCGATAG

* F: forward primer, R: reverse primer. The "m" prior to a primer name indicates it is for a mouse gene; "r" for a rat gene; "mr" for both mouse and rat gene. Primer name including "clone" indicates it is for gene cloning.

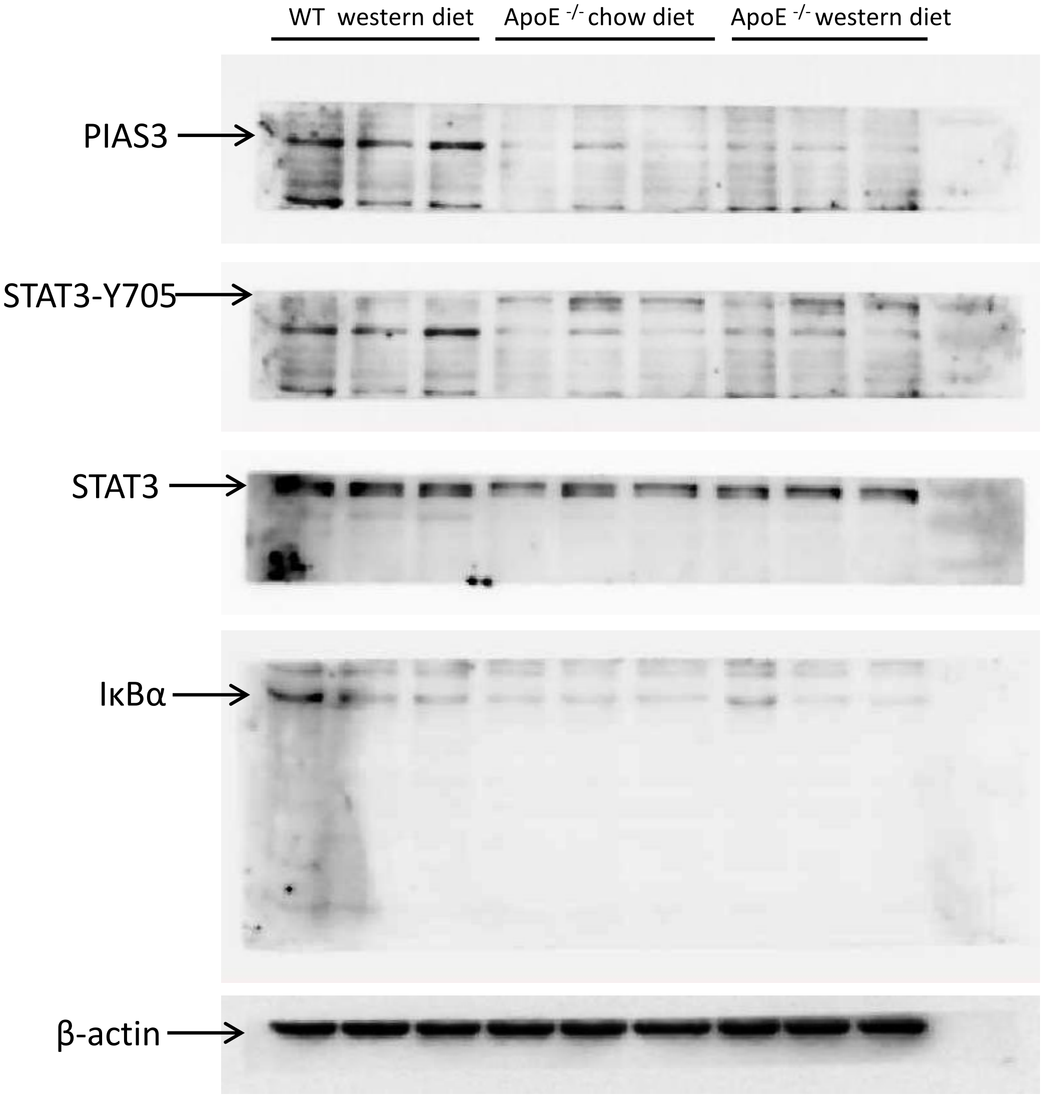

Supplementary Figure S1. **Full-length blots of Figure 1E.** For detecting PIAS3, IκBα and β-actin on the PVDF membrane at the same time, the PVDF membrane was cut into three pieces according to prestained protein marker (Cat. No. 180-6003, Tanon), after transfer. Then, the small pieces of membrane were probed with antibodies against PIAS3, IκBα and β-actin, respectively. To detect proteins with close or same molecular weight (such as PIAS3, STAT3-Y705 and STAT3), the probed-membrane was stripped by stripping buffer (Cat. No. 46430, Thermo Fisher Scientific) after detected by an antibody, and then re-probed with other antibody again. The blots of target protein were indicated with an arrow.

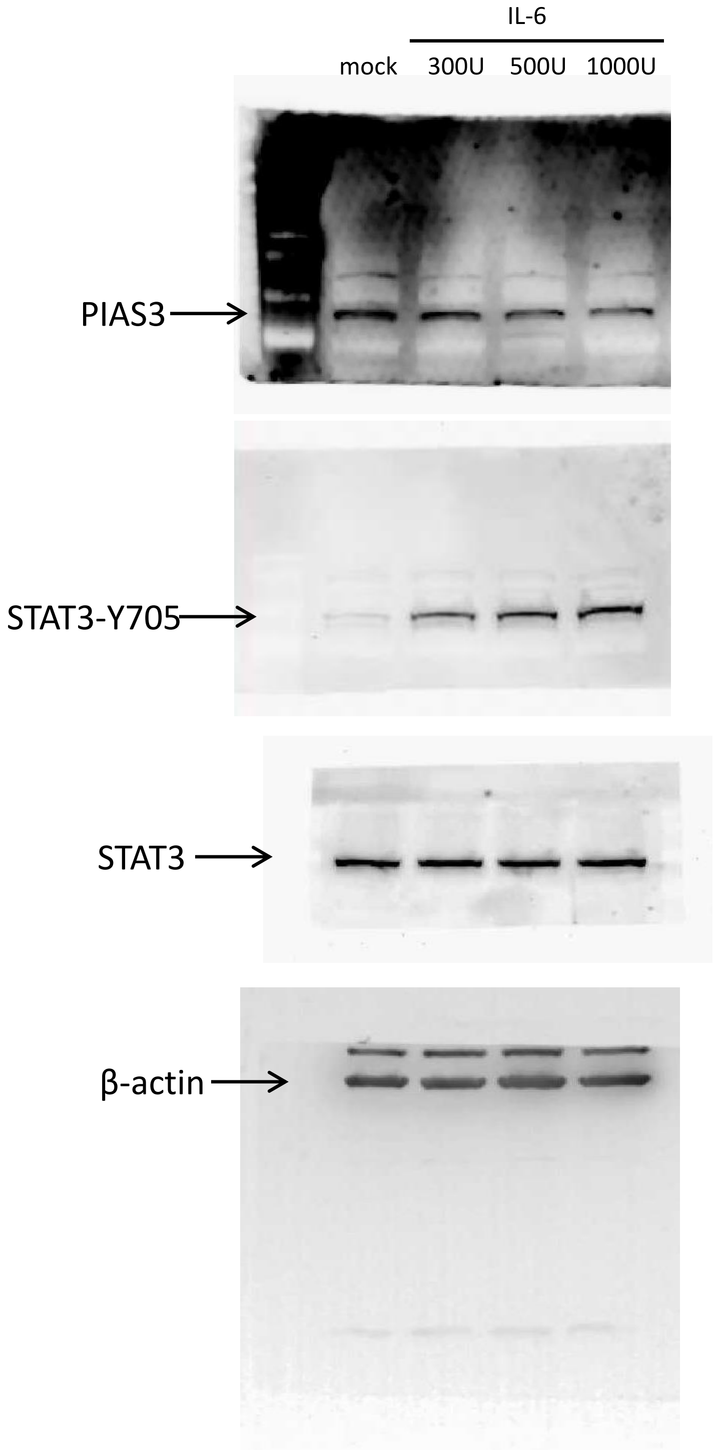

Supplementary Figure S2. **Full-length blots of Figure 2B.** For detecting PIAS3 and β-actin on the PVDF membrane at the same time, the PVDF membrane was cut into two pieces according to prestained protein marker (Cat. No. 180-6003, Tanon), after transfer. Then, the membranes were probed with antibodies against PIAS3 and β-actin, respectively. To detect proteins with close or same molecular weight (such as PIAS3, STAT3-Y705 and STAT3), the probed-membrane was stripped by stripping buffer (Cat. No. 46430, Thermo Fisher Scientific) after detected by an antibody, and then re-probed with other antibody again. The blots of target protein were indicated with an arrow.

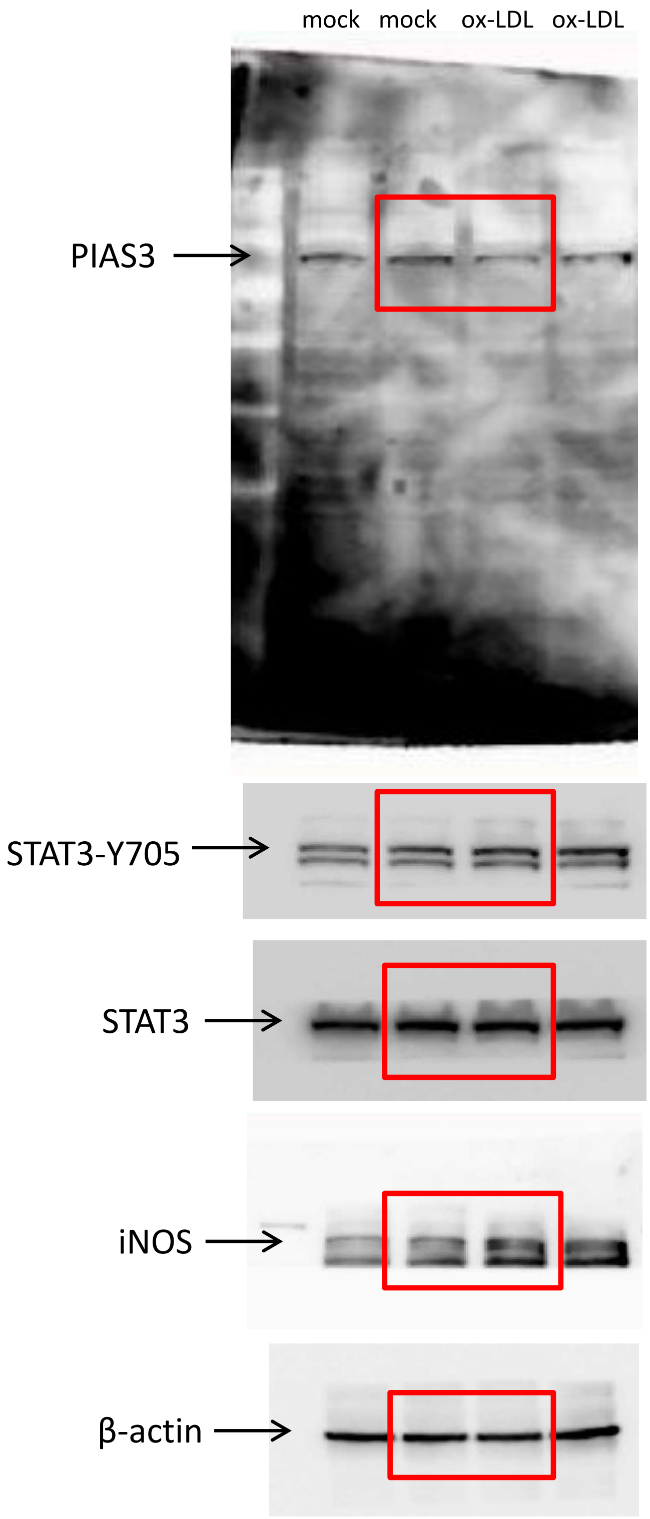

Supplementary Figure S3. **Full-length blots of Figure 2D.** The PVDF membrane was probed with antibody against PIAS3 firstly. Then, the membrane was stripped by stripping buffer (Cat. No. 46430, Thermo Fisher Scientific). For detecting STAT3-Y705, iNOS and β-actin on the PVDF membrane at the same time, the stripped-membrane was cut into three pieces according to prestained protein marker (Cat. No. 180-6003, Tanon). Then, the small pieces of membrane were re-probed with antibodies against STAT3-Y705, iNOS and β-actin, respectively. To detect STAT3 protein, the probed-membrane was stripped again after detected by anti-STAT3-Y705 antibody, and then re-probed with anti-STAT3 antibody. The blots of target protein were indicated with an arrow. The cropped blots, displayed in the Figure 2D, were shown in red rectangle.

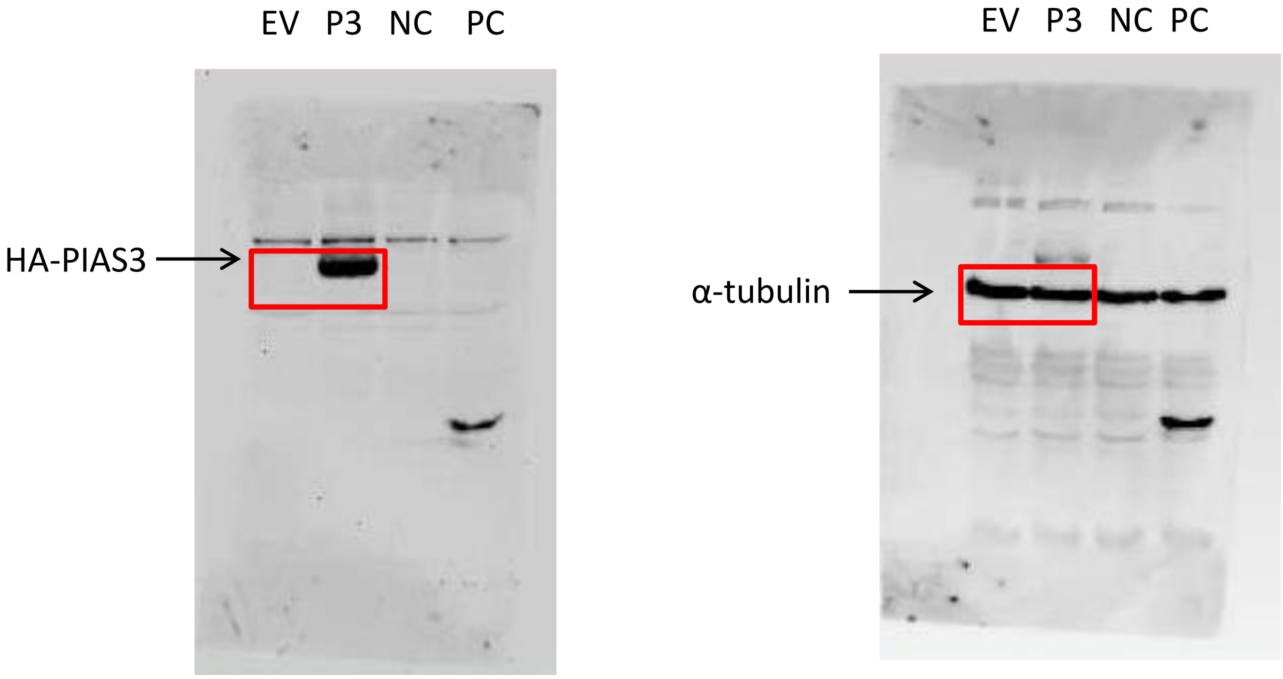

Supplementary Figure S4. **Full-length blots of Figure 3A.** To detect the pCAGEN-HA-PIAS3 plasmid expression, the PVDF membrane was probed with anti-HA antibody. Then, the membrane was stripped by stripping buffer (Cat. No. 46430, Thermo Fisher Scientific), followed by re-probed with anti-α-tubulin antibody. The blots of target protein were indicated with an arrow. The cropped blots, displayed in the Figure 3A, were shown in red rectangle. EV: empty vector transfected cells; P3: pCAGEN-HA-PIAS3 transfected cells; NC: negative control (cells without transfection); PC: positive control (cells transfected with a known HA-tagged plasmid).

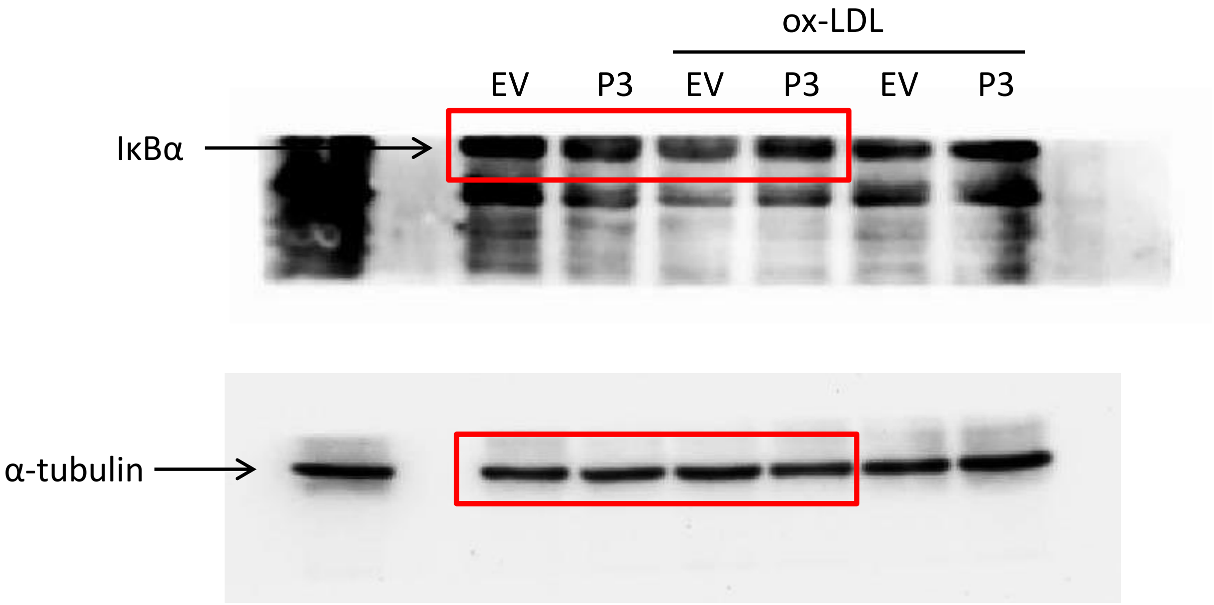

Supplementary Figure S5. **Full-length blots of Figure 3B.** For detecting IκBα and α-tubulin on the PVDF membrane at the same time, the PVDF membrane was cut into two pieces according to prestained protein marker (Cat. No. 180-6003, Tanon), after transfer. Then, the membranes were probed with antibodies against IκBα and α-tubulin, respectively. The blots of target protein were indicated with an arrow. The cropped blots, displayed in the Figure 3B, were shown in red rectangle.

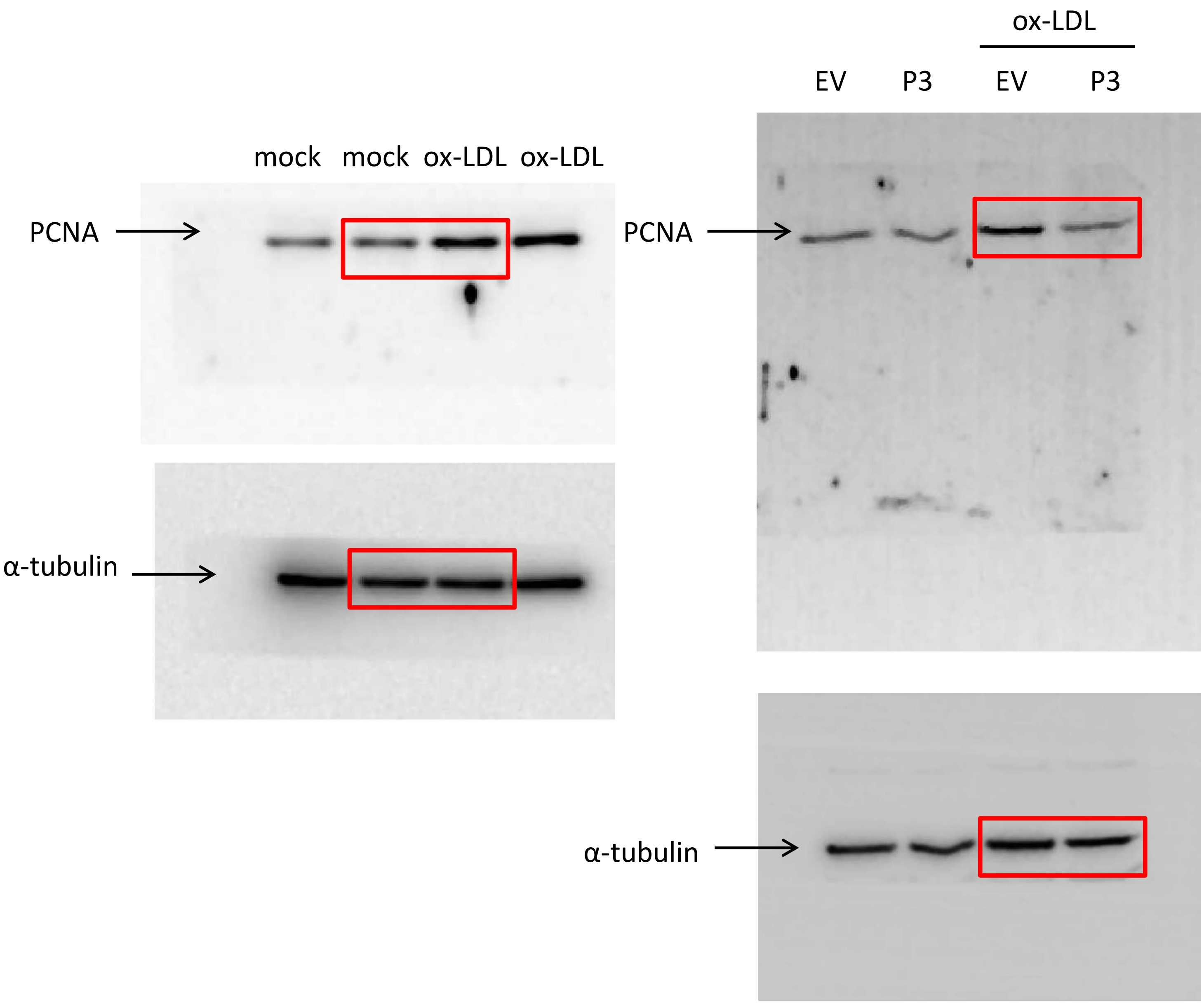

Supplementary Figure S6. **Full-length blots of Figure 5B.** For detecting PCNA and α-tubulin on the PVDF membrane at the same time, the PVDF membrane was cut into two pieces according to prestained protein marker (Cat. No. 180-6003, Tanon), after transfer. Then, the membranes were probed with antibodies against PCNA and α-tubulin, respectively. The blots of target protein were indicated with an arrow. The cropped blots, displayed in the Figure 5B, were shown in red rectangle. The left two images and the right two images are obtained from different experiments.
